# Correlation between osteoarthritis of the atlantoaxial facet joint and a high‐riding vertebral artery

**DOI:** 10.1186/s12891-021-04275-9

**Published:** 2021-05-03

**Authors:** Tomoaki Shimizu, Masao Koda, Tetsuya Abe, Yosuke Shibao, Mamoru Kono, Fumihiko Eto, Kousei Miura, Kentaro Mataki, Hiroshi Noguchi, Hiroshi Takahashi, Toru Funayama, Masashi Yamazaki

**Affiliations:** Department of Orthopedic Surgery, Faculty of Medicine, University of Tsukuba, 1-1-1 Tennodai, 305-8575 Tsukuba, Ibaraki Japan

**Keywords:** High riding vertebral artery, Prevalence, Subaxial cervical spine surgery, C2 pedicle screw, Posterior instrumentation surgery, Osteoarthritis of C1-2 facet joints

## Abstract

**Background:**

A high-riding vertebral artery (HRVA) is an intraosseous anomaly that narrows the trajectory for C2 pedicle screws. The prevalence of a HRVA is high in patients who need surgery at the craniovertebral junction, but reports about HRVAs in subaxial cervical spine disorders are limited. We sought to determine the prevalence of HRVAs among patients with subaxial cervical spine disorders to elucidate the potential risk for VA injury in subaxial cervical spine surgery.

**Methods:**

We included 215 patients, 94 were with a main lesion from C3 to C7 (subaxial group) and 121 were with a main lesion from T1 to L5 (thoracolumbar group). A HRVA was defined as a maximum C2 pedicle diameter of < 3.5 mm on axial CT. The sex, age of patients, body mass index (BMI), osteoarthritis of the atlantoaxial (C1-2) facet joints, and prevalence of a HRVA in the 2 groups were compared and logistic regression was used to identify the factors correlated with a HRVA.

**Results:**

The patients in the subaxial group were younger than those in the thoracolumbar group, but their sex and BMI did not differ significantly between the 2 groups. The mean osteoarthritis grade of the C1-2 facet joints of patients in the subaxial group was significantly higher than that in those in the thoracolumbar group. A HRVA was found in 26 patients of 94 (27.7 %) in the subaxial group and in 19 of 121 (15.7 %) in the thoracolumbar group. The prevalence of a HRVA in the subaxial group was significantly higher and osteoarthritis of C1-2 facet joints correlated significantly with a HRVA.

**Conclusions:**

The prevalence of a HRVA in patients with subaxial cervical spine disorders is higher than in those without and osteoarthritis of the C1-2 facet joints is correlated with a HRVA.

## Background

Cervical pedicle screws (CPSs) have enabled us to perform rigid internal fixation [1]. In particular, PSs for the axis (C2 PSs) have been used more frequently because they are the most feasible and reliable anchor for posterior instrumentation surgery in the cervical spine. However, they have to be applied carefully because of the possibility of injury to the vertebral artery (VA) caused by the misposition of a CPS, which could develop into serious complications, such as cerebellar infarction or brain stem infarction [2]. Thus, it is very important to evaluate the course of the VA preoperatively and select appropriate instruments for surgery.

A high riding vertebral artery (HRVA) is an intraosseous anomaly that unusually courses too medially, too posteriorly, and/or too superiorly at the isthmus of the C2 vertebra, resulting in narrowing of the trajectory for C2 PS. There is a significantly higher risk of VA injury by C2 PS insertion in patients with a HRVA [3]. To date there have been several studies of the prevalence of a HRVA. In studies using cadavers, the prevalence of a HRVA was found as 11.7–20.0 % [4, 5]; in studies targeting patients with cervical spine disorders the prevalence was found as 14.5–31.0 % [3, 6]; and in those without cervical spine disorders the prevalence was found as 10.1–16.5 % [7, 8]. The prevalence of a HRVA is especially high in surgery at the craniovertebral junction [6, 9]. Thus, it is possible that the prevalence of a HRVA is different depending on the level of spinal pathology. However, there are scant reports of a HRVA in patients with subaxial cervical spine disorders despite that posterior fixation is frequently performed for the subaxial cervical spine including C2. The primary objective of this study was to compare the prevalence of HRVAs among patients with subaxial cervical spine disorders with that among those with thoracolumbar spine disorders to elucidate the potential risk for VA injury in subaxial cervical spine surgery.

It is known that rheumatoid arthritis (RA) causing deformity of the C1-2 facet joint is a risk factor for a HRVA [10–12]. Tomasz et al. reported that the estimated prevalence of a HRVA in those with RA was 42.9 % and significantly higher than that in those without RA [12]. However, we often detect a HRVA when we perform a posterior cervical spine surgery for those without RA. Therefore, the secondary objective of the present study was to clarify the risk factors for a HRVA other than RA. We focused on the deformity of the C1-2 facet joints and investigated the correlation between the degenerative change of the C1-2 facet joints and a HRVA.

## Materials and methods

**Fig. 1:**
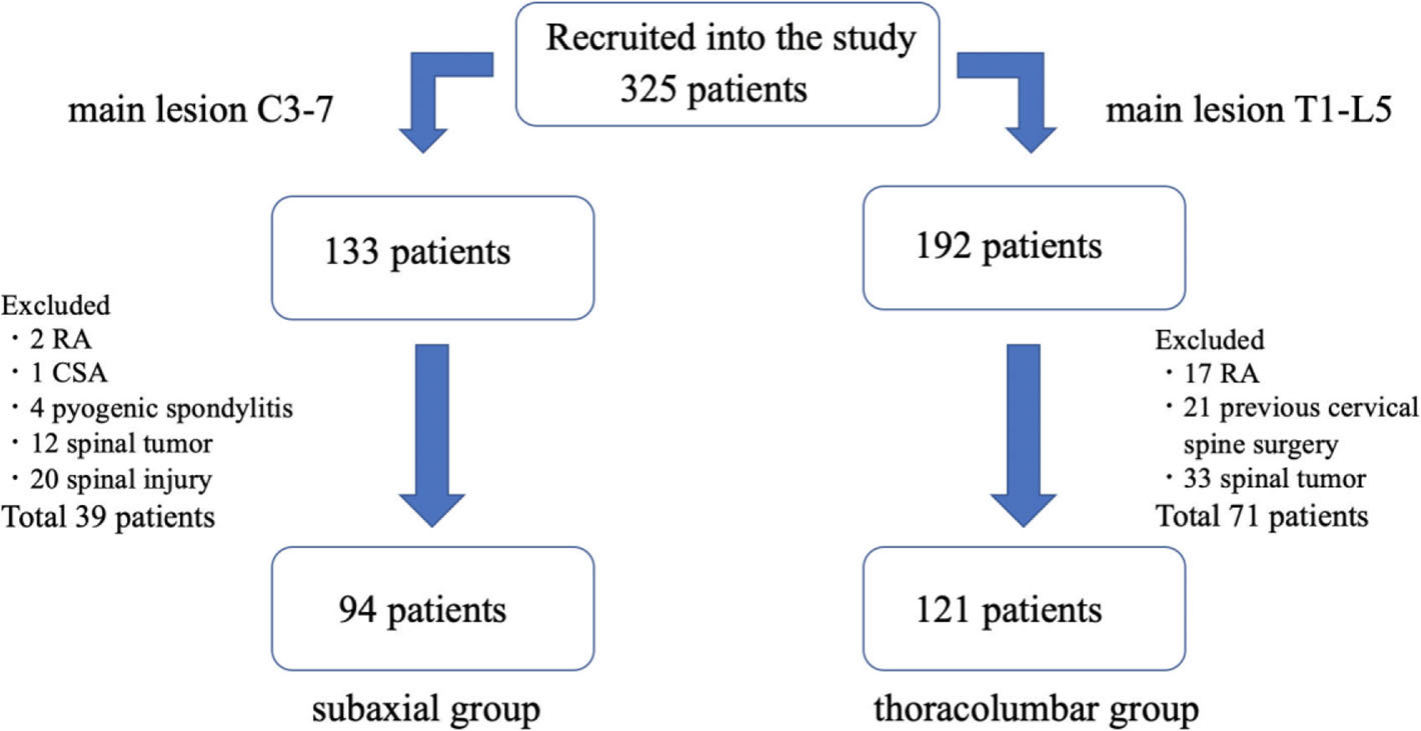
Patient flow chart. RA: rheumatoid arthritis. CSA: congenital skeletal anatomy

We analyzed the medical records of 325 consecutive patients who underwent subaxial cervical spine surgery (subaxial group) and who underwent myelography for thoracolumbar disorders (thoracolumbar group) at our institute from December 2012 to June 2020. The study was approved by our institutional review board and informed consent was obtained from all participants. The subaxial group included those with their main lesion from C3 to C7, and the thoracolumbar group included those with their main lesion from T1 to L5. In both groups, those with rheumatoid arthritis and congenital skeletal anatomy were excluded because these conditions are already known as risk factors for a HRVA. In addition, those with pyogenic spondylitis, spinal tumor, spinal injury, and patients with previous cervical spine surgery were excluded (39 patients in the subaxial group and 71 patients in the thoracolumbar group, Fig. 1).

Ultimately, the subaxial group included 94 patients (67 men, 27 women, mean age 61.9 years) and the thoracolumbar group included 121 patients (71 men, 50 women, mean age 67.1 years).

The subaxial group included 48 patients with ossification of the posterior longitudinal ligament (OPLL), 38 with cervical spondylosis, 3 with cervical spondylotic radiculopathy, 3 with cervical spine kyphosis, and 2 with cervical spondylotic amyotrophy. The surgical method in the subaxial group was posterior fixation including C2 (46 patients), posterior fixation above C3 (25 patients), anterior fusion (16 patients), and posterior laminoplasty without fixation (7 patients). In 46 patients who underwent posterior fixation including C2, the instruments used for C2 were as follows: PSs were applied bilaterally to 20 patients, unilaterally to 2 patients, PSs and laminar screws bilaterally to 21 patients, pars screws and laminar screws bilaterally to 2 patients, and laminar screws bilaterally to 1 patient.

The thoracolumbar group included 82 patients with lumbar spinal canal stenosis, 17 with degenerative lumbar kyphoscoliosis, 15 with thoracic ossification of the yellow ligament (OYL), and 7 with thoracic spondylotic myelopathy.

**Fig. 2:**
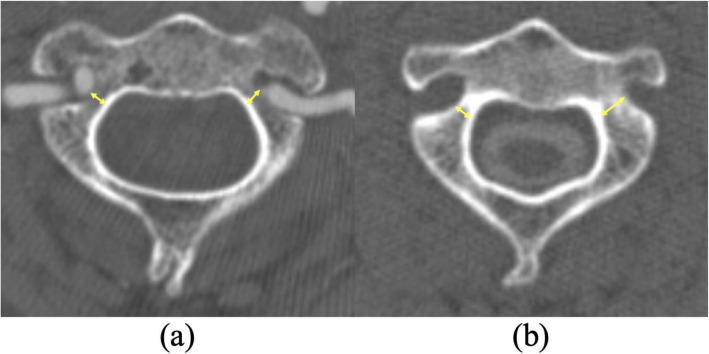
Pedicle diameter of the axis (C2) measured on axial computed tomography (CT). **a** Axial CT angiography. The pedicle diameter on both sides was <3.5 mm. **b** Axial CT myelography. The pedicle diameter on the left side was >3.5 mm, but <3.5 mm on the right side

We performed preoperative 3-dimensional computed tomographic angiography (3D CTA) for all the patients in the subaxial group and CT myelography for all the patients in the thoracolumbar group. For all the patients in each group, we searched for HRVAs in axial slices (1 mm thick) that were along the orthogonal horizontal plane (Fig. 2). We defined a HRVA as a maximum C2 pedicle diameter of < 3.5 mm on the axial image, because the minimal diameter of the commonly used screw is 3.5 mm.

**Fig. 3:**
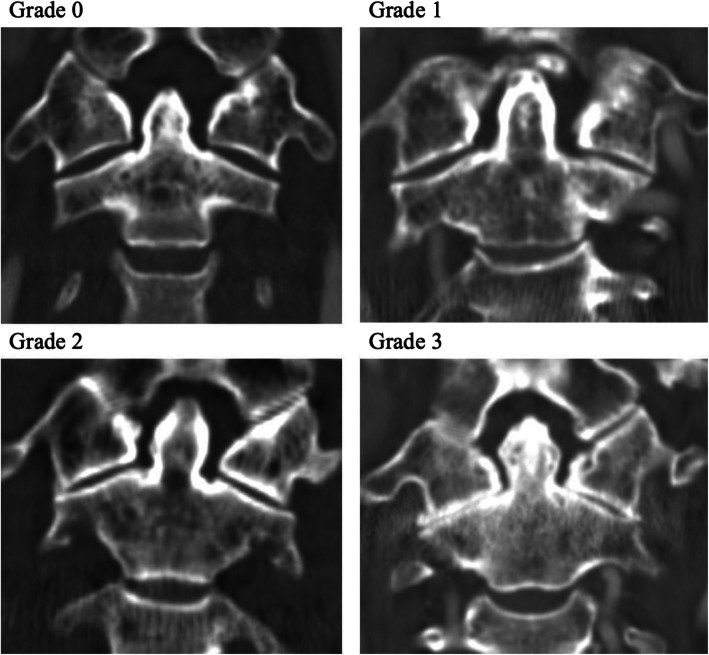
Grading scale for osteoarthritis of the atlantoaxial (C1-2) facet joints. Grade 0 = normal. Grade 1 = joint space narrowing. Grade 2 = narrowing plus sclerosis. Grade 3 = severe osteoarthritis with narrowing, sclerosis, and osteophytes

In addition, we evaluated osteoarthritis of the atlantoaxial (C1-2) facet joints in the coronal plane of CT of all the patients in each group. Assessment of facet joint osteoarthritis was conducted with a grading scale as described previously [13, 14]. Grade 0 indicates a normal facet joint, grade 1 shows joint space narrowing, grade 2 shows narrowing and sclerosis of facet joint, and grade 3 shows narrowing, sclerosis and osteophytes (Fig. 3).

The sex, age of patients, body mass index (BMI), osteoarthritis grade of the C1-2 facet joints, and prevalence of a HRVA in the 2 groups were compared using an unpaired Student *t* test, a Pearson χ^2^ test, and a Mann–Whitney *U* test as appropriate. Logistic regression was used to identify the risk factor for a HRVA. Age, sex, BMI, and osteoarthritis grade of the C1-2 facet joints were added as independent variables for multiple logistic regression analysis. All the analyses were performed using IBM SPSS Statistics for Windows (version 26.0) and statistical significance was considered established at *p* < 0.05.

The instruments used for C2 pedicles with a HRVA in the subaxial group were analyzed. For all the patients in the subaxial group, we analyzed neurovascular injury as a complication of surgery derived from screw insertion.

## Results

The age of the patients differed significantly between the 2 groups, but the sex ratio and BMI did not differ significantly between the 2 groups (Table 1).

Of 94 patients in the subaxial group, 17 presented with grade 0 (18.1 %) osteoarthritis of the atlantoaxial (C1-2) facet joints, 24 with grade 1 (25.5 %), 45 with grade 2 (47.9 %), and 8 with grade 3 (8.5 %). Of 121 patients in the thoracolumbar group, 24 presented with grade 0 (19.8 %) osteoarthritis of the atlantoaxial (C1-2) facet joints, 46 with grade 1 (38.0 %), 47 with grade 2 (38.8 %), and 4 with grade 3 (3.3 %). When we compared the grades of osteoarthritis between the subaxial group and the thoracolumbar group, the mean grade in the subaxial group was significantly higher than that the thoracolumbar group (*p* = 0.037; Table 1).

A HRVA was detected in 45 (20.9 %) of the 215 patients. Of 94 patients in the subaxial group, 26 (27.7 %) had a HRVA. Of 121 patients in the thoracolumbar group,19 (15.6 %) had a HRVA. The prevalence of a HRVA in patients in the subaxial group was significantly higher than it was in the thoracolumbar group (*p* = 0.033; Table 1).

**Table 1:** Data summary for patients in the subaxial and thoracolumbar groups

	Subaxial group (94 patients)	Thoracolumbar group (121 patients)	*p*
Sex^a^	Male 67	Male 71	0.06
	Female 27	Female 50	
Age^a^	61.9 ± 11.1	67.1 ± 10.2	0.001^*^
BMI^a^	25.6 ± 5.8	24.8 ± 4.5	0.29
Diagnosis	cervical OPLL 48, cervical spondylosis 38, cervical spondylotic radiculopathy 3, cervical spine kyphosis 3, cervical spondylotic amyotrophy 2	lumbar spinal canal stenosis 82, degenerative lumbar kyphoscoliosis 17, thoracic OYL 15, thoracic spondylotic myelopathy 7	
OA grade of the C1-2 facet joints^a^	Grade 0 17	Grade 0 24	0.037^*^
	Grade 1 24	Grade 1 46	
	Grade 2 45	Grade 2 47	
	Grade 3 8	Grade 3 4	
HRVA^a^	26 cases (27.7%)	20 cases (15.6%)	0.033^*^

^a^Sex and the prevalence of a high-riding vertebral artery (HRVA) were compared using a Pearson χ^2^ test. Age and BMI were compared using an unpaired Student *t* test. OA grade of the C1-2 facet joints was compared using a Mann–Whitney *U* test. *p < 0.05

*BMI* Body mass index, *OPLL* Ossification of the posterior longitudinal ligament, *OYL* Ossification of the yellow ligament, *OA* Osteoarthritis

Multiple logistic regression indicated that osteoarthritis of the C1-2 facet joints was a significant risk factor for a HRVA (odds ratio 1.66, 95 % confidence interval 1.05–2.63, *p* = 0.031; Table 2).

**Table 2:** Multiple logistic regression of risk factors for a high-riding vertebral artery (HRVA)

Variable^a^	Odds ratio	95% confidence interval	*p*
sex (female: male)	1.19	0.59–2.34	0.64
age	0.98	0.95–1.02	0.36
BMI	0.98	0.91–1.05	0.53
OA grade of the C1-2 facet joints	1.82	1.17–2.84	0.008^*^

^a^For this analysis, male = 0 and female = 1. *p < 0.05

*BMI* Body mass index, *OA* Osteoarthritis

For those with a HRVA in the subaxial group (31 sides of 26 patients), 9 sides had a laminar screw inserted, and in 22 sides no screw was inserted (Table 3). No PS was applied to those with a HRVA. No neurovascular injury derived from screw insertion occurred in any patient.

**Table 3:** Data for instruments used for the C2 pedicle in patients with a HRVA (31 sides of 26 patients)

Instrument		
Pedicle screw	0	
Laminar screw	9	
Pars screw	0	
No insertion	Anterior fusion	9
	Only decompression	1
	Fixation above C3	10
	Avoid insertion	2
Total	31	

## Discussion

In the present study, which excluded those with RA, the prevalence of a HRVA in the subaxial group was 27.7 % and that in the thoracolumbar group was 15.6 %, as consistent with findings of previous studies. The prevalence of a HRVA in patients in the subaxial group was significantly higher than that in those in the thoracolumbar group. This means that a HRVA is more frequent in patients with a subaxial cervical spine disorder than in normal individuals, and that the prevalence of a HRVA might be lower in patients with thoracolumbar disorders.

While it is already known that RA and Down syndrome, which cause deformity of the C1-2 facet joint, are risk factors for a HRVA, the present study suggests that subaxial cervical spine disorder is also associated with the presence of a HRVA. Vanek et al. reported that female sex and age > 70 years were significant risk factors for a HRVA [15], which was not confirmed in the present study, nor was BMI found to be a risk. Rather, logistic regression using the present data indicated that osteoarthritis of a C1-2 facet joint is a significant risk factor for a HRVA. Degenerative change of the C1-2 facet joints may lead to bone deformation and thinning of the C2 isthmus as found in those with RA; therefore, the prevalence of a HRVA in subaxial cervical spine disorders in which C1-2 facet joints tend to degenerate is more frequent than in normal individuals. As such, we should be vigilant for a HRVA when we perform surgery not only for those with RA, Down syndrome, and with the main lesion at the craniovertebral junction, but also in those with a subaxial cervical spine disorder.

Tomasz et al. reported that systemic, periarticular, and local osteoporosis could affect the formation of a HRVA in those with RA [11]. According to this hypothesis and the results of this study, a HRVA could be considered to have an acquired nature. Although, a HRVA is more frequent in those with congenital skeletal anatomy [6]. We were unable to conclude whether an acquired factor or a congenital factor is of more importance in forming of a HRVA, and a HRVA could have both an acquired and a congenital nature.

The definition of HRVA in this study using the axial plane of CT differs from the widely accepted definition. In general, a HRVA is usually defined with sagittal plane of CT as a C2 isthmus height of ≤5mm and/or internal height of ≤2 mm [12] and the definition of HRVA in this study was more of a “narrow pedicle”. However, Maki et al. reported that a medially-shifted VA precluded safe C2 PS insertion and that axial plane of CT should be evaluated to avoid VA injury before inserting C2 PS [16]. Thus, we followed their definition because “medially-shifted” VA demands more attention than a “high-riding” VA when we insert a C2 PS in clinical practice.

In general, C2 PSs are considered safer than subaxial (C3–C6) PSs in patients without a HRVA because the trajectory of a C2 PS can be confirmed directly and the insertion angle for a C2 PS is significantly less mediolaterally than the angle for a subaxial PS. Therefore, C2 PSs tend to be used more frequently for posterior fixation surgery. In the present study, more than half of the patients who underwent posterior surgery in the subaxial group (46 of the 78 cases) needed fixation including C2. However, insertion of C2 PSs in patients with a HRVA bears a considerable risk of VA injury [17]. Yoem et al. reported that among the C2 PSs inserted for patients with a HRVA, about half violated the VA groove [3], which indicates that C2 PSs are not appropriate for patients with a HRVA. We did not use a C2 PS in any patient with a HRVA, but used a laminar screw instead or avoided inserting screws entirely. As a result, we had no neurovascular injury as a complication of surgery in these patients. Moreover, Salunke et al. reported a “C2 subfacetal screw” that was inserted directly into the C2 body while safeguarding the VA that was dissected from the medial border of the C2 transverse foramen [18]. This screw insertion technique may be an alternative for those with a HRVA. When instrumentation for C2 is needed for patients who will undergo subaxial cervical spine surgery, we should look carefully for a HRVA using preoperative CT, and apply instruments other than PSs in those with a HRVA.

There are several limitations to our study. First, the patients examined in this study were all Japanese admitted to a single university hospital. It may not be possible to widely generalize the results of our study to patients of other ancestry. Second, the sample size of our study was relatively small.

## Conclusion

The prevalence of a HRVA in patients with subaxial cervical spine disorders is higher than in those without and osteoarthritis of the C1-2 facet joints correlates significantly with a HRVA.

## Data Availability

The datasets generated during and analyzed during the current study are available from the corresponding author on reasonable request.

## Abbreviations

CPS: Cervical pedicle screw
HRVA: High riding vertebral artery
OPLL: Ossification of the posterior longitudinal ligament
OYL: Ossification of the yellow ligament
BMI: Body mass index
RA: Rheumatoid arthritis

## References

[1] Abumi K, Ito H, Taneichi H (1994). Transpedicular screw fixation for traumatic lesions of the middle and lower cervical spine: description of the techniques and preliminary report. J Spinal Disord.

[2] Wright NM, Lauryssen C (1998). Vertebral artery injury in C1-2 transarticular screw fixation: results of a survey of the AANS/CNS section on disorders of the spine and peripheral nerves. J Neurosurg.

[3] Yeom JS, Buchowski JM, Kim HJ (2013). Risk of vertebral artery injury: comparison between C1-C2 transarticular and C2 pedicle screws. Spine J.

[4] Mandel IM, Kambach BJ, Petersilge CA (2000). Morphologic considerations of C2 isthmus dimensions for the placement of transarticular screws. Spine.

[5] Madawi AA, Casey AT, Solanki GA (1997). Radiological and anatomical evaluation of the atlantoaxial transarticular screw fixation technique. J Neurosurg.

[6] Yamazaki M, Okawa A, Furuya T (2012). Anomalous vertebral arteries in the extra- and intraosseous regions of the craniovertebral junction visualized by 3-dimensional computed tomographic angiography: analysis of 100 consecutive surgical cases and review of the literature. Spine (Phila Pa 1976).

[7] Wakao N, Takeuchi M, Nishimura M (2014). Vertebral artery variations and osseous anomaly at the C1-2 level diagnosed by 3D CT angiography in normal subjects. Neuroradiology.

[8] Wajanavisit W, Lertudomphonwanit T, Fuangfa P (2016). Prevalence of high-riding vertebral artery and morphometry of C2 pedicles using a novel computed tomography reconstruction technique. Asian Spine J.

[9] Yamazaki M, Okawa A, Hashimoto M (2008). Abnormal course of the vertebral artery at the craniovertebral junction in patients with Down syndrome visualized by three-dimensional CT angiography. Neuroradiology.

[10] Miyata M, Neo M, Ito H (2008). Rheumatoid arthritis as a risk factor for a narrow C2 pedicle: 3D analysis of the C-2 pedicle screw trajectory. J Neurosurg Spine.

[11] Tomasz K, Jagoda C, Leszek S (2020). Risk of the high-riding variant of vertebral arteries at C2 is increased over twofold in rheumatoid arthritis: a meta-analysis. Neurosurgical Rev.

[12] Tomasz K, Bartomiej P, Jagoda C, et al. Prevalence of high-riding vertebral artery: a meta-analysis of the anatomical variant affecting choice of craniocervical fusion method and its outcome. World Neurosurg. 2020;143:e474-e481 10.1016/j.wneu.2020.07.182 32750514

[13] Pathria M, Sartoris DJ, Resnick D (1987). Osteoarthritis of the facet joints: accuracy of oblique radiographic assessment. Radiology.

[14] Thorsten J, James G, Stefarn MZ (2013). Lumbar facet joint arthritis is associated with more coronal orientation of the facet joints at the upper lumbar spine. Radiol Res Pract.

[15] Vanek P, Bradac O, Lacy P (2017). Vertebral artery and osseous anomalies characteristic at the craniocervical junction diagnosed by CT and 3D CT angiography in normal Czech population: analysis of 511 consecutive patients. Neurosurg Rev.

[16] Maki S, Koda M, Iijima Y (2016). Medially-shifted rather than high-riding vertebral arteries preclude safe pedicle screw insertion. J Clin Neurosci.

[17] Park JH, Lee JB, Lee HJ (2019). Accuracy evaluation of placements of three different alternative C2 screws using the freehand technique in patients with high riding vertebral artery. Medicine (Baltimore).

[18] Salunke P, Singh A, Karthigeyan M (2020). Technique of C2 Subfacetal Screw in Patients with High-Riding Vertebral Artery. World Neurosurg.

